# A Penetrating Stab Wound of the Perianal Area Causing a Combined Rectal and Bladder Injury: One Case Report

**DOI:** 10.1155/2012/137281

**Published:** 2012-07-16

**Authors:** Mohammed Fadl Tazi, Abdelhak Khallouk, Youness Ahallal, Omar Riyach, Jalal Eddine El Ammari, Mohammed Jamal El Fassi, Moulay Hassan Farih

**Affiliations:** ^1^Department of Urology, HassanII Hospital University Center, Fez, Morocco; ^2^Faculté de Médecine et de Pharmacie de Fès BP/1893, Km 2,200 Route de Sidi Harazem, Fès, Morocco

## Abstract

Although the management of either isolated rectal or bladder injury is no more controversial, their combined effect and their optimal management has been seldom reported in the English literature. From a case report of a 45-year-old male who was found to have a combined bladder and rectal injury secondary to a stab wound of the perianal area, the authors develop a diagnostic and therapeutic algorithm for the management of this uncommon trauma.

## 1. Introduction 

A missed rectal injury can have devastating consequences. Although isolated bladder injury produce little morbidity or mortality, an overlooked combined rectal-and-bladder injury can produce serious morbidity and can even lead to death. To our knowledge, this is the first paper dealing with the management of combined bladder-and-rectal injury secondary to penetrating stab wound of the perianal area. Following a case report together with a review of the literature, this paper aims at reminding the clinical and therapeutic features of this rare trauma.

## 2. Case Presentation

**Figure 1:**
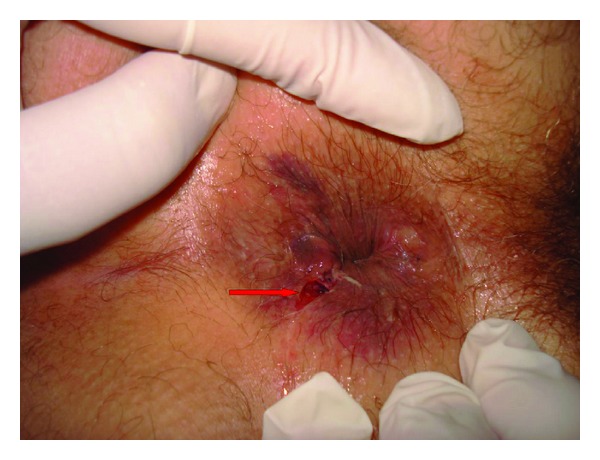
Perineal examination showing a wound located laterally to the anal opening.

**Figure 2:**
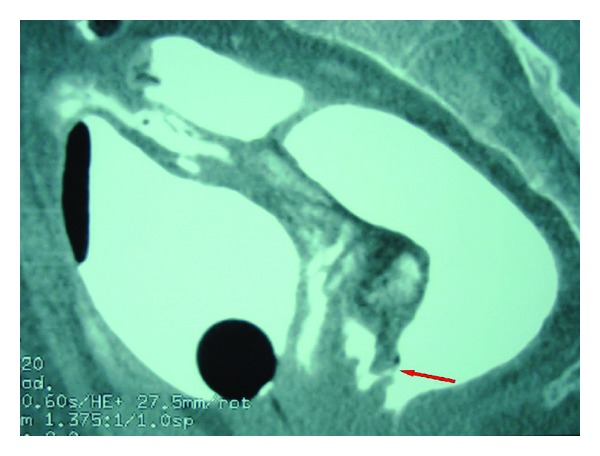
Cystogram CT showing contrast extravasation with anterior rectal wall defect.

**Figure 3:**
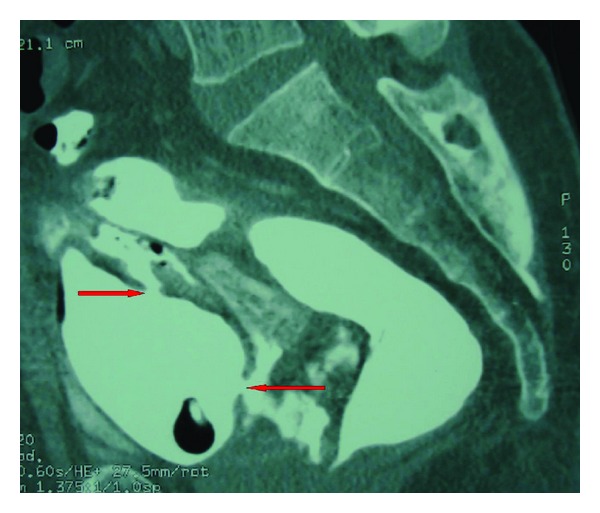
Cystogram CT showing contrast extravasation with 2 bladder defects.

A 45-year-old male Moroccan patient presented to the emergency department with gross rectal bleeding and hematuria secondary to a stab wound to the perianal area. The patient was admitted four hours after his knife stabbing. On physical examination, the patient looked well and was stable hemodynamically. He had mild suprapubic tenderness and the rectal examination revealed evidence of rectal bleeding together with a 2 cm wound, located 1 cm laterally to the anal opening (Figure 1). A proctosigmoidoscopy performed on the patient revealed two defects of the posterior and the anterior walls of the rectum. An emergency cystogram CT was done through a urethral catheter under screening, and the rectum was also filled with the contrast in order to improve its evaluation. This clearly showed an extraperitoneal contrast extravasation associated with an anterior rectal wall defect (Figure 2) and two bladder wall defects located in the posterior wall and the dome of the urinary bladder (Figure 3). The CT showed no abnormalities in the nearby organs. The rectal injury treatment consisted of distal rectal washout, transanal rectal wound repair that was protected by a diverting colostomy and the bladder injury was managed by a urethral catheter drainage. Three months later, the colostomy was reversed without other sequela. The urethral catheter was left in place for 30 days, and the patient was put on oral broad spectrum prophylactic antibiotics.

A retrograde cystogram was done 30 days later and showed no further evidence of extravasation. After a one-year followup, the patient is healthy and totally asymptomatic.

## 3. Discussion

Combined rectal-and-bladder injury associated with penetrating stab wound of the perianal area are very rare. The standard management of penetrating rectal trauma consists of perioperative antibiotics, a diverting colostomy, and presacral drainage. While providing optimal results in isolated rectal trauma, this management scheme is inadequate in combined penetrating rectal-and-bladder injuries.

Most publications dealing with combined rectal-and-bladder injuries due to penetrating injury are mostly reported in lesions of the buttock and are related to gunshots. When a stabbed patient presents gross rectal bleeding and hematuria, which are strong indicators of colorectal and bladder injuries, proctoscopy and cystogram should be performed [1]. To diagnose the bladder injury, it's mandatory to perform a retrograde static cystogram or a CT cystography. For the highest diagnostic accuracy, the bladder must be distended by the instillation of at least 350 cc of contrast medium. Indeed, Carroll and McAninch found that 100% accuracy could be achieved if 350 cc contrast was instilled [2]. When a retrograde static cystogram is performed, the area behind the bladder should be imaged after the instilled contrast has been drained to obviate missing extravasation obscured by the intravesical contrast [3]. CT cystography is increasingly being performed as an alternate method of diagnosing bladder rupture and it has been shown to be as accurate as conventional cystography [1]. However, CT cystography has several advantages over plain film cystography: it is rapid and is easily performed at the same time as other CT studies, it is less affected by overlying bone fragments, and it is more sensitive to the detection of small amounts of intraperitoneal or extraperitoneal fluid [4]. Thus, when it is decided to perform a CT scan for other lesions, CT cystography must be preferred to retrograde cystography.

Franko et al. recommend to treat all the rectal injuries when they are associated with genitourinary injury with diverting colostomy, rectal wound repair, presacral drainage, and rectal washout [5]. Diverting the fecal stream is the main aspect of the treatment as it reduces the risk of rectovesical fistulas development. Indeed, it is unclear, whether nonrepaired rectal defects combined with bladder injuries are associated with an increased incidence of complications. However, Franko et al. showed an increase in complications' rate in the absence of rectal wound repair in combined injuries [5]. Maull et al. had managed their patients' colon injuries by closure of the perforation and diverting proximal colostomy and the bladder injuries were treated by oversewing the perforation site [6]. The most common reported postoperative complication was fever; no patient developed sepsis though, despite gross urinary and fecal contamination [6]. Tuggle and Huber reported one case of a rectoprostatic fistula and two cases of rectovesical fistulas in the absence of rectal wound repair [7].

Because of their close anatomical proximity, such wounds are more likely to cause sepsis; we believe therefore that the use of presacral drainage, rectal wound repair, rectal washout, and diverting colostomy are mandatory; and the complications' risk is amplified if any of these measures is omitted [5].

In our case, we decided to perform a transanal rectal wound repair protected by a diverting colostomy without presacral drainage, and to manage the bladder injury with prolonged Foley catheterization (i.e., 30 days) with a very good result.

## 4. Conclusion

Because penetrating rectal trauma is uncommon, its management can be particularly challenging especially if associated with bladder injury. Rectal bleeding and hematuria are the main symptoms. Proctoscopy may define the nature and site of penetration and should be performed upon all patients together with a cystogram CT which is the most accurate imaging examination to assess bladder injuries. The management of this rare combination consists of surgical treatment of the rectal wound. We have showed that the bladder injury could be managed conservatively with prolonged Foley catheterization.
